# Secretory Expression and Application of Antilipopolysaccharide Factor 3 in *Chlamydomonas reinhardtii*

**DOI:** 10.3390/bioengineering10050564

**Published:** 2023-05-08

**Authors:** Yaohui Ou, Huilin Zhuang, Ruoyu Chen, Danqiong Huang, Chaogang Wang

**Affiliations:** 1 Guangdong Technology Research Center for Marine Algal Bioengineering, College of Life Sciences and Oceanography, Shenzhen University, Shenzhen 518060, China; 2 Shenzhen Engineering Laboratory for Marine Algal Biological Development and Application, College of Life Sciences and Oceanography, Shenzhen University, Shenzhen 518060, China; 3 Laboratory of Marine Bioresource & Eco-Environmental Science, College of Life Sciences and Oceanography, Shenzhen University, Shenzhen 518060, China; 4 Guangdong Provincial Key Laboratory for Plant Epigenetics, College of Life Sciences and Oceanography, Shenzhen University, Shenzhen 518060, China

**Keywords:** anti-lipopolysaccharide factor 3, *Chlamydomonas reinhardtii*, secretory expression, antibacterial activity

## Abstract

Anti-lipopolysaccharide factor is a class of antimicrobial peptides with lipopolysaccharide-binding structural domains, which has a broad antimicrobial spectrum, high antimicrobial activities, and broad application prospects in terms of the aquaculture industry. However, the low yield of natural antimicrobial peptides and their poor expression activity in bacteria and yeast have hindered their exploration and utilization. Therefore, in this study, the extracellular expression system of *Chlamydomonas reinhardtii*, by fusing the target gene with the signal peptide, was used to express anti-lipopolysaccharide factor 3 (ALFPm3) from *Penaeus monodon* in order to obtain highly active ALFPm3. Transgenic *C. reinhardtii* T-JiA2, T-JiA3, T-JiA5, and T-JiA6, were verified using DNA-PCR, RT-PCR, and immunoblot. Additionally, the IBP1-ALFPm3 fusion protein could be detected not only within the cells but also in the culture supernatant. Moreover, the extracellular secretion containing ALFPm3 was collected from algal cultures, and then its bacterial inhibitory activity was analyzed. The results showed that the extracts from T-JiA3 had an inhibition rate of 97% against four common aquaculture pathogenic bacteria, including *Vibrio harveyi, Vibrio anguillarum, Vibrio alginolyticus,* and *Vibrio parahaemolyticus*. The highest inhibition rate of 116.18% was observed in the test against *V. anguillarum*. Finally, the minimum inhibition concentration (MIC) of the extracts from T-JiA3 to *V. harveyi, V. anguillarum, V. alginolyticus,* and *V. parahaemolyticus* were 0.11 μg/μL, 0.088 μg/μL, 0.11 μg/μL, and 0.011 μg/μL, respectively. This study supports the foundation of the expression of highly active anti-lipopolysaccharide factors using the extracellular expression system in *C. reinhardtii*, providing new ideas for the expression of highly active antimicrobial peptides.

## 1. Introduction

In recent years, the abuse of antibiotics in the aquaculture industry has increased drug-resistant pathogens and created super bacteria, which seriously affected human production and life [1,2,3]. To overcome this situation, several governments have ordered a ban on the addition of antibiotics in feed production. Therefore, there is an urgent need to find antibiotic alternatives that are friendly to humans and the environment in order to meet the requirement of the aquaculture industry. Antimicrobial peptides are peptides consisting of 5–300 amino acids and are widely distributed in various organisms [4,5,6]. Antimicrobial peptides are attractive because of their powerful antimicrobial activity, decreased susceptibility to drug resistance, and high safety [7,8]. Among them, anti-lipopolysaccharide factors (ALFs) [9], which were first discovered in the blood cells of *Limulus polyphemus*, exhibit unique antibacterial activity by binding and disrupting bacterial cell walls and cell membranes with their lipopolysaccharide structural domains. Currently, various ALFs have been identified in *Penaeus monodon* [10,11]. Studies have shown that anti-lipopolysaccharide factor 3 (ALFPm3) has a broader antibacterial spectrum and antimicrobial activity; hence, it is one of the best candidates for antibiotic replacement [11,12].

The hetero-expression of antimicrobial peptides is mainly in *Escherichia coli* and yeast. However, using *E. coli* to express antimicrobial peptides faces several problems, such as difficulty in terms of expression and the easy formation of inclusion bodies [11,13,14]. The expression of antimicrobial peptides using yeast also suffers from excessive protein glycosylation, which reduces the activity of the antimicrobial peptide [15,16]. In addition, *E. coli* and yeast require more nutrients and energy when farmed on a larger scale, leading to increased production costs, which is not conducive to the development of a low-carbon economy. As a single-celled eukaryotic green algae, *Chlamydomonas reinhardtii* has the advantages of a short growth cycle, high photosynthetic efficiency, and a well-developed genetic transformation system. In addition, its expression products can be directly applied to aquaculture, such as fish and shrimp, with low cost and convenience, becoming an excellent “green factory” for antimicrobial peptide expression [17]. Previously, antimicrobial peptides have been successfully expressed within the cell of *C. reinhardtii* [18,19,20,21]. Considering the future application in aquaculture, the secreted expression of antimicrobial peptides into the extracellular space would be a better choice. Unfortunately, studies related to the secretion of antimicrobial peptides in microalgae have not been reported.

The secretion of recombinant proteins into the extracellular matrix will facilitate their subsequent application and extraction. In addition, glycosylation modifications in the secretory expression pathway could maintain protein stability and function [22,23]. Previously, an endogenous signal peptide named ARS1 was used to accomplish extracellular expression to enhance the expression of Xylanase1 (Xyn1) in *C. reinhardtii*. The transformed algal cells were able to target monomeric xylanase for secretion. Despite differences in the productivity of Xyn1 obtained intracellularly and extracellularly (possibly due to the modification of Xyn1 in the secretory pathway), purified secretory and cytoplasmic Xyn1 showed similar activity [22]. Later on, more than 2000 signal peptides could be identified with the software SignalP 4.0 and nine of them were confirmed to promote the secretion of exogenous proteins in *C. reinhardtii* [24]. As one of them, the signal peptide Ice-binding protein 1 (IBP1), derived from a psychrophilic Antarctic alga [25], has been successfully used in *C. reinhardtii cc1690* to promote the intracellular secretion of mcherry fluorescent protein and performed better than other signal peptides [24]. In the present study, aiming to produce antimicrobial peptides that can be secreted into the extracellular compartment, ALFPm3 derived from *P. monodon* was fused to IBP1. The strong native promoter psaD was used to drive the efficient heterologous expression of IBP1-ALFPm3 in *C. reinhardtii*. Finally, we obtained transgenic algae that successfully synthesized ALFPm3 and secreted it into culture media, which could effectively inhibit the growth of *Vibrio harveyi, Vibrio anguillarum, Vibrio alginolyticus,* and *Vibrio parahaemolyticus*. It is noted that the inhibition rate of protein extracts from culture media of transgenic algal cells was significantly higher than that of ampicillin, indicating the superiority of the obtained ALFPm3 we designed in this study on the bacteria inhibition. This study will support the foundation study for expressing highly active anti-lipopolysaccharide factors using the extracellular expression system in *C. reinhardtii*, providing new ideas concerning the expression of highly active antimicrobial peptides. 

## 2. Materials and Methods

### 2.1. Strains and Culture Conditions

Cell-wall-deficient *Chlamydomonas reinhardtii* JUV was purchased from the Chlamydomonas Resource Center (USA) and cultured in tris-phosphate (TAP) liquid or TAP agar solid medium containing 100 µg/mL ampicillin (Biosharp, Hefei, China). Algal cells were grown in a growth chamber with a temperature of 22–25 °C and a light intensity of 90 μE/m^2^·s. Transgenic algae were selected and cultured on TAP liquid or agar solid medium containing both 100 µg/mL ampicillin and 10 µg/mL zeocin (Invitrogen, Carlsbad, CA, USA).

The bacteria used in this study, including *Vibrio harveyi*, *Vibrio anguillarum*, *Vibrio alginolyticus*, and *Vibrio parahaemolyticus* were gifted by Prof. Lishi He from the South China Sea Fisheries Research Institute, Chinese Academy of Fisheries Sciences, and cultured with LB medium at 37 °C and 200 rpm in rotary shaker (Shanghai Zhicheng Analytical Instrument Manufacturing Co., ZHWY-2102C, Shanghai, China).

### 2.2. Plasmid Construction and Algae Transformation

**Figure 1:**
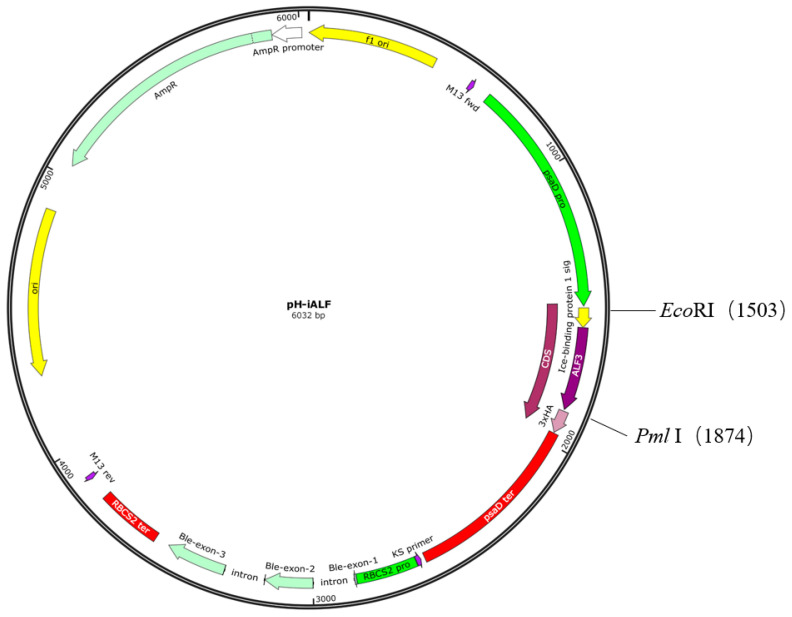
Schematic diagram of plasmid pH-iALF.

Anti-lipopolysaccharide factor ALF3 from *Penaeus monodon* (ALFPm3) (Genbank accession number JQ256520) was optimized according to the codon preference of the *C. reinhardtii* nuclear genome. The signal peptide of IBP1 (23aa: MPSSSMKLFAALLIACMAQTSMA) was linked at the N-terminal of ALFPm3, and a 3×HA tag was linked at the N-terminal of ALFPm3 to obtain the fragment of IBP1-ALFPm3, which was then inserted into the vector pESVH constructed in our laboratory to form the plasmid of pH-iALF (Figure 1). The constructed plasmid of pH-iALF was transferred into *E. coli* Top 10 for proliferation.

The nuclear transformation of *C. reinhardtii* was processed using the glass bead method, according to the literature [26]. The transformants named T-JiA were screened and maintained on TAP agar plates containing 100 µg/mL ampicillin and 10 µg/mL zeocin under the conditions of 22 °C and 90 μE/m^2^·s continuous light. The number of algal colonies was recorded, and the transformation frequency was calculated based on the following equation: Transformation frequency = number of algal colonies/total number of recipient cells.

### 2.3. Genomic PCR and RT-PCR Analysis

The positive transgenic *C. reinhardtii* were identified through the use of genomic PCR and RT-PCR analysis. For the genomic PCR, genomic DNA was extracted from the algal cells cultured to the mid-logarithmic phase using the Ultra DNA isolation kit (Beibei Biotechnology Co., Zhengzhou, China). The 20 µL PCR reaction system contained 2 µL of DNA extract, a psaD-P/psaD-T primer pair (Table 1), and 2× M5 HiPer plus Taq HiFi PCR mix (Mei5bio, Beijing, China). The PCR program was 95 °C for 3 min, followed by 35 cycles of 94 °C for 25 s, 58 °C for 25 s, and 72 °C for 40 s, and then a final extension of 72 °C for 5 min. For the RT-PCR, the total RNA was extracted from the collected algal cells using the RNA fast 200 kit (Fastagen, Shanghai, China). A final 1 µg of the total RNA was used for the synthesis of the cDNA strands using Hifair^®^ Ⅲ 1st Strand cDNA Synthesis SuperMix for qRCR (gDNA digester plus) (Yeasen, Shanghai, China), according to the manual. Primer pairs Fi/ALF3R2 targeting IBP1-ALFPm3 and actin-F/actin-R (Table 1) targeting β-actin were used. The RT-PCR program was 95 °C for 3 min, followed by 35 cycles of 95 °C for 30 s, 58 °C for 30 s, and 72 °C for 40 s, and then a final extension of 72 °C for 5 min. Finally, all of the PCR products were analyzed using 1.5% agarose gel electrophoresis. 

### 2.4. Protein Extraction and Immunoblot Analysis

For intracellular protein extraction, the algal cells at the mid-logarithmic stage were collected using centrifugation and the extraction was accomplished according to the reference [12]. For extracellular protein extraction, the culture solution was collected through centrifugation and then freeze-dried to obtain a lyophilized powder, which was then solubilized with 1× PBS (Beyotime Biotechnology, Shanghai, China). The protein concentration was determined using the BCA Protein Concentration Assay Kit (Beyotime Biotechnology, Shanghai, China). For the immunoblot analysis, 20–30 μg of total protein was separated using 12.5% SDS-PAGE (GenScript, Piscataway, NJ, USA) and transferred to a PVDF membrane (Merck, Rowe, NJ, USA). The membrane was subsequently washed 3 times with 1×TBST solution and incubated with Anti-HA.11 Epitope Tag Antibody (1:2000) (Biolegend, San Diego, CA, USA) overnight, and finally incubated with Anti-mouse IgG, HRP-linked Antibody (1:2000) (Cell Signaling Technology, Boston, MA, USA). Chemiluminescent signals were developed using BeyoECL Moon (Beyotime Biotechnology, Shanghai, China) as detected through Odyssey^®^ Fc (Gene Company Limited, Hong Kong, China).

### 2.5. Antibacterial Test

The confirmed transgenic *C. reinhardtii* T-JiA successfully synthesized and secreted ALFPm3 were cultured to the mid-logarithmic stage at 25 °C and a continuous light intensity of 90 μE/m^2^·s for the antibacterial test. The culture supernatant was obtained through the use of centrifugation, freeze-dried, and finally solubilized with 1×PBS (Beyotime Biotechnology, Shanghai, China) to obtain protein extracts. Four common aquaculture pathogenic bacteria: *Vibrio harveyi, Vibrio alginolyticus, Vibrio anguillarum*, and *Vibrio parahaemolyticus* were used to test the antibacterial function of protein extracts. Before the assay, the pathogenic bacteria were inoculated into 3 mL of the fresh LB liquid medium and cultured overnight at 37 °C with 200 rpm in a rotary shaker (Shanghai Zhicheng Analytical Instrument Manufacturing Co., ZHWY-2102C, Shanghai, China). Overnight cultures were subsequently inoculated into new fresh LB media at a rate of 1:100, and new cultures were incubated at 37 °C and 200 rpm for 1 h. Finally, the new cultures were then diluted 1000 times using fresh LB liquid medium for subsequent experiments. In a sterile 96-well plate, 150 μL of the bacterial culture was mixed with 50 μL of the protein extracts. The mixture was maintained at 37 °C for 24 h, and its absorbance values at 600 nm were measured every 2 h. As a positive control, 2 mg/mL of ampicillin was used instead of the protein extracts. As a negative control, the protein extracts from the wild-type of *C. reinhardtii JUV* were used. The growth rate was represented as the ΔOD600, which referred to the changes in absorbance at different time points with the absorbance values at 0 h. The bacterial inhibition rate was calculated by the following equation:

$$Inhibition\;rate\;(\%)=\frac{\left(\Delta OD600\;negative\;group-\Delta OD600\;experimental\;group\right)}{\Delta OD600\;negative\;group}\times 100\%$$

### 2.6. Minimum Inhibition Concentration (MIC) Assay

The method used to obtain MIC was carried out according to reference [12]. Here, 1×PBS was used for the gradient dilution of the protein extracts. In a sterile 96-well plate, 50 μL of the bacterial culture was mixed with 50 μL of the protein extract samples at different dilutions, respectively. A total of 2 mg/mL of ampicillin was used as a positive control, and the bacterial culture was used as a negative control. The growth rate was represented as ΔOD600, which referred to the change in OD600 at 12 h to the OD600 values at 0 h. The different dilutions were plotted as horizontal coordinates, and ΔOD600 was plotted as vertical coordinates to obtain the minimum inhibitory concentration of the extracellular protein extraction.

### 2.7. Statistical Analysis

Each experiment contained three independent replicates. GraphPad Prism 8 was used for the statistical analysis and to generate graphs. In this paper, a *t-*test was used to test the statistical differences between the different treatment groups.

## 3. Results

### 3.1. Design of ALFPm3 Expression Cassette

**Figure 2:**
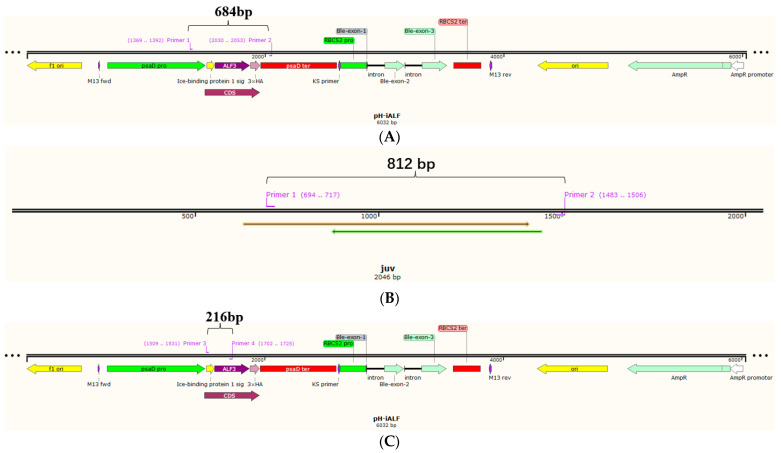
The characterization of IBP1-ALFPm3 expression cassette. Primers 1 is psaD-P. Primers 2 is psaD-T. Primers 3 is Fi. Primers 4 is ALF3R2. (**A**,**C**) IBP1-ALFPm3 expression cassette. The IBP1-ALFPm3 fused gene was overexpressed under the drive of psaD promoter and terminator. (**B**) The native psaD gene in *C. reinhardtii*.

The ALFPm3 gene was optimized based on the codon preference of *C. reinhardtii* nuclear genome, and the GC content of the optimized ALFPm3 gene increased from 56% to 68%. The IBP1-ALFPm3 fused gene was inserted into the restriction endonuclease sites of *Eco*RI and *Bst*EII on the pESVH vector to obtain pH-iALF (Figure 1). The IBP1-ALFPm3 fused gene was expressed under the drive of the psaD promoter and the psaD terminator (Figure 2), which could overexpress the target gene under strong light. 

### 3.2. Confirmation of Transgenic C. reinhardtii

pH-iALF plasmids were introduced into *C. reinhardtii* JUV using the glass bead method, and transformants were selected using a TAP plate containing 100 µg/mL ampicillin and 10 µg/mL zeocin. According to the number of colonies grown, the transformation frequency was 2.2 × 10^−5^, and the *C. reinhardtii* transformant was renamed T-JiA.

**Figure 3:**
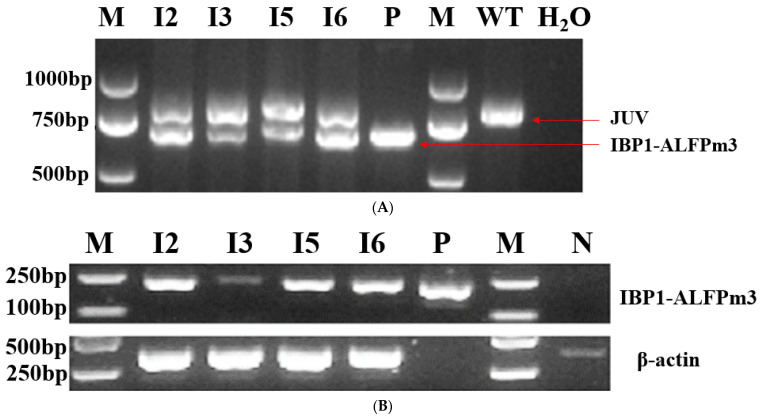
Gel electrophoresis of the PCR products for the molecular identification of transgenic *C. reinhardtii* T-JiA. (**A**) Genomic PCR analysis of transgenic *C. reinhardtii* T-JiA. JUV: PCR products from *psaD* gene in C. reinhardtii. IBP1-ALFPm3: PCR products from the IBP1-ALFPm3 expression cassette. (**B**) RT-PCR analysis of Transgenic *C. reinhardtii* T-JiA. M: DL 2000 DNA ladder marker. P: positive control, pH-iALF plasmid DNA as the PCR template. WT: wild-type *C. reinhardtii*, as the negative control. H_2_O: no template control. I2, I3, I5, I6: four different transgenic *C. reinhardtii* lines.

PsaD-P is an endogenous promoter in microalgae, which is responsible for initiating *PsaD* nuclear gene encoding, an associated protein located on the proximal stromal side of the photosystem I complex in chloroplasts [27]. Therefore, both wild-type *C. reinhardtii* and positive transgenic *C. reinhardtii* will detect the corresponding gene fragments in the genomic PCR with psaD-P/psaD-T primer sets. The genomic PCR results showed that only an 812 bp fragment was detected in wild-type *C. reinhardtii*, as expected. An additional 684 bp fragment was presented in the positive transgenic *C. reinhardtii* as the positive control, indicating that the IBP1-ALFPm3 fused gene was successfully inserted into the genome of *C. reinhardtii* JUV (Figure 3A). The RT-PCR results showed that a 216 bp fragment was observed in positive transformants as the positive control, meaning that the IBP1-ALFPm3 fusion gene was successfully transcribed in *C. reinhardtii* (Figure 3B).

### 3.3. Secretion of IBP1-ALFPm3 Fusion Protein 

**Figure 4:**
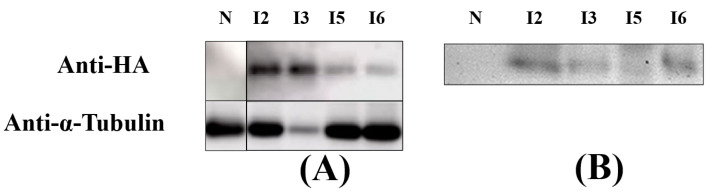
Immunoblot analysis of T-JiA. (**A**) Immunoblot analysis of IBP1-ALFPm3 fusion protein in the protein extracts from the algal cells of T-JiA. (**B**) Immunoblot analysis of IBP1-ALFPm3 fusion protein in the protein extracts from the culture media of T-JiA. N: wild-type *C. reinhardtii* JUV served as the negative control. I2–I6: transgenic algae T-JiA lines I2–I6 detected with anti-HA antibody. Anti-α-tubulin was an internal reference, incubated with an anti-α-tubulin antibody.

With the beneficial 3× HA-tag located at the C-terminal of IBP1-ALFPm3 fused protein, immunoblotting was performed to identify the presence of fused protein in protein extracts from the algal cells and the culture media of transgenic *C. reinhardtii* T-JiA. An expected band was detected in the intracellular protein extracted from I2, I3, I5, and I6 but not detected in that from non-transgenic *C. reinhardtii*, indicating that IBP1-ALFPm3 fusion protein was synthesized and accumulated within the algal cells (Figure 4A). Moreover, IBP1-ALFPm3 fusion protein could be detected in the culture supernatant of I2, I3, I5, and I6 while there was detection in wild-type of *C. reinhardtii* JUV, indicating that the IBP1-ALFPm3 fusion protein was secreted into the media as expected (Figure 4B). Though the IBP1-ALFPm3 fusion proteins could be detected in the culture supernatant, many of them remained within cells. Additionally, significant differences in terms of secretion efficiency were observed among the transformants. 

### 3.4. Extracellular Protein Extracts from T-JiA Showed High Antibacterial Activity

**Figure 5:**
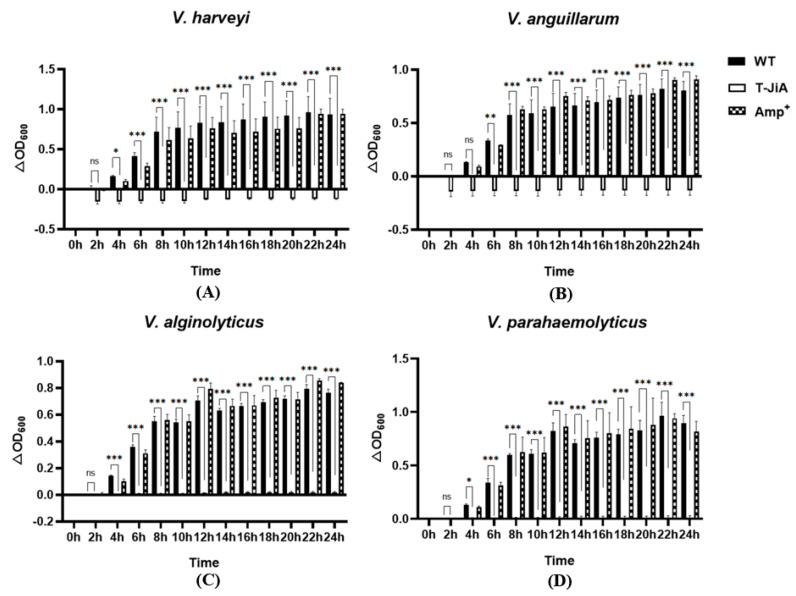
The effect of the protein extracts containing ALFPm3 from the culture medium of I3 on the growth of common aquaculture pathogenic bacteria, including *V. harveyi* (**A**), *V. anguillarum* (**B**), *V. alginolyticus* (**C**), and *V. parahaemolyticus* (**D**). WT: culture supernatant of non−transgenic *C. reinhardtii JUV*. T-JiA: culture supernatant of T-JiA I3. Amp^+^: Ampicillin (2 mg/mL). *, significance at the level of 0.05. **, significance at the level of 0.01. ***, significance at the level of 0.001. ns, no significance at the level of 0.05.

To test the activity of the ALFPm3 secreted into the culture medium of T-JiA, the protein extracts from the culture medium were prepared and tested. The protein concentration in the extracts ranged from 0.46 to 0.91 μg/μL. The antibacterial activity was tested against the pathogenic bacteria of *Vibrio harveyi*, *Vibrio anguillarum*, *Vibrio alginolyticus*, and *Vibrio parahaemolyticus*, which commonly exist in aquaculture. The results showed that I3 has the best antibacterial effect, and the protein extracts significantly inhibited the growth of *V. harveyi*, *V. anguillarum*, *V. alginolyticus*, and *V. parahaemolyticus* within 24 h. The inhibition rate of IBP1-ALFPm3 against four aquaculture pathogenic bacteria was over 97%, with the highest inhibition rate of 116.18% against *V. anguillarum*. Although the concentration of ampicillin used in this study was 2 mg/mL, which was nearly 20 times higher than its working concentration of 100 μg/mL, the inhibition rate against those four bacteria was under 30.27% at 8 h. Therefore, the inhibition effect of IBP1-ALFPm3 was much better than that of ampicillin (2 mg/mL), indicating that the IBP1-ALFPm3 secreted into the culture media possessed a strong antibacterial activity (Figure 5). 

### 3.5. The MIC of Extracellular Protein Extracts from T-JiA

**Figure 6:**
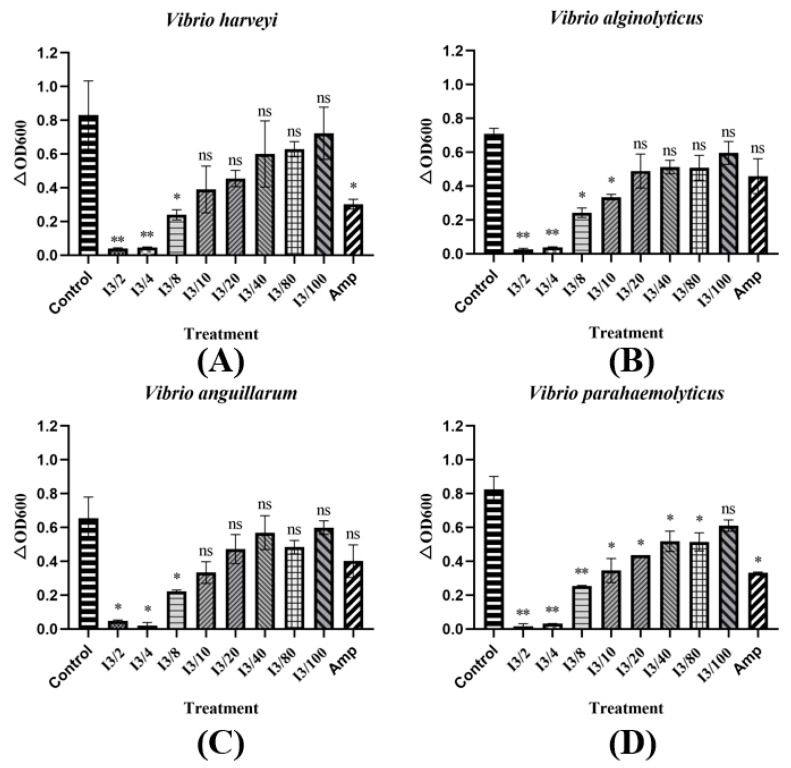
The MIC of extracellular protein extracts in relation to common aquaculture pathogenic bacteria, including *V. harveyi* (**A**)*, V. anguillarum* (**B**), *V. alginolyticus* (**C**), and *V. parahaemolyticus* (**D**). Control: LB culture. Amp: Ampicillin (2 mg/mL). I3/2: 0.44 μg/μL. I3/4: 0.22 μg/μL. I3/8: 0.11 μg/μL. I3/10: 0.088 μg/μL, I3/20: 0.044 μg/μL, I3/40: 0.022 μg/μL, I3/80: 0.011 μg/μL, I3/100: 0.0088 μg/μL. Each group was compared with the control group for significant differences. *, significance at the level of 0.05. **, significance at the level of 0.01. ns, no significance at the level of 0.05.

Due to the outstanding inhibition ability of T-JiA I3 in the antibacterial test, its minimum inhibition concentration (MIC) was studied. The secreted protein of T-JiA I3 was diluted in a gradient and then used in a MIC test. Our results showed that the inhibition ability in terms of *Vibrio* decreased as the concentration of extracellular protein decreased. The MIC of *V. harveyi, V. anguillarum, V. alginolyticus,* and *V. parahaemolyticus* were 0.11 μg/μL, 0.088 μg/μL, 0.11 μg/μL, and 0.011 μg/μL, respectively. Moreover, the extracellular protein extracts showed the strongest activity against *V. parahaemolyticus* at 0.011 μg/μL, which was 10 times that of *V. harveyi* and *V. alginolyticus* (Figure 6).

## 4. Discussion

Although antimicrobial peptides have the advantages of high antimicrobial activity and decreased susceptibility to resistance, they are naturally expressed at low levels in organisms. Therefore, heterologous expression at high yields with no loss of activity is an important means of antimicrobial peptide production. Currently, most AMP production is hosted by *E. coli* and yeast [28]. However, both have some shortcomings that are difficult to overcome. The expression of the antimicrobial peptide human beta defensin 2 (HBD2) with *E. coli* under specific induction conditions can increase its expression by an order of magnitude, but such harsh conditions are not suitable for industrial production [29]. It is also important to note that *E. coli* is not a suitable host for the direct expression of high concentrations of antimicrobial peptides because they are toxic for the growth and development of *E. coli* [30]. Although this problem can be solved by fusing the protein, the target protein also needs to be purified and enzymatically digested using a Ni^2+^ affinity column before it can be applied, increasing the cost of production [31,32,33]. In addition, there is the unknown risk of degradation related to exogenous proteases, challenging the prokaryotic expression systems applied for broad usage [33]. Although antimicrobial peptides do not cause harmful effects on yeast growth and reproduction, their expressed proteins are often modified through the over-methylation in the extracellular secretory pathway. The overexpression can also lead to protein misfolding and trigger host cell stress. This will reduce the production efficiency of the target protein [15,16,34]. Yeast often requires the addition of methanol to induce the maximum action of its promoter, which not only increases production costs but also leads to toxicity [35]. As a unicellular eukaryotic green microorganism, *C. reinhardtii* has matured in genetic transformation technology and has been used in the production of various proteins, such as enzymes, antibodies, and vaccines [22,36,37]. *C. reinhardtii* has many advantages, such as rapid growth, protein post-translational processing capabilities, the ability to photoautotrophy using low-cost media, and no release of metabolites and endotoxins that are unfriendly to the environment or humans, and it is a good platform for expressing recombinant proteins [28,38,39]. Nowadays, microalgae scale culture technology has been widely used, which easily enables the production of target products at scale with reduced cost, and the edibility of microalgae will greatly increase the potential for future applications.

In previous studies, *C. reinhardtii* was used to produce antimicrobial peptides such as ToAMP4, Mytichitin-CB, and Bacteriocin LS2. The expressed products were effective in inhibiting the growth of Gram-negative and positive bacteria [18,19,20]. These studies demonstrate that *C. reinhardtii* can be used as a platform to produce active antimicrobial peptides. However, the intracellular expression of antimicrobial peptides also means that the protein needs to be purified by ultrasonic lysis, and this reduces the continuity of production. Extracellular expression simplifies the isolation of exogenous gene expression products, and the target protein can be accurately post-transcriptionally modified. A report showed that the signal peptide of IBP1 could mediate the extracellular expression of mCherry fluorescent protein in *C. reinhardtii.* The result showed that the total culture fluorescence value of the algal strain exceeded 10,000 RFU, showing good protein activity. The supernatant to whole-culture fluorescence value ratio was over 75% [24]. Noting that the effect of IBP1 was superior to most of the signal peptides in their study, IBP1 was used in this study to develop the secretion of ALFPm3 in *C. reinhardtii*. Through immunoblot analysis, we found that ALFPm3 existed in both cell lysates and the culture supernatants. The obtained extracellular protein extract concentrations ranged from 0.46 to 0.91 μg/μL. The high concentration of the extracts verified the effectiveness and efficiency of the IBP1-mediated extracellular secretion of ALFPm3.

In addition to the good yield, the obtained ALFPm3 through the secretory pathway also has excellent bacterial inhibitory activity. According to previous studies, ALFPm3 extracted from algal cells has an inhibitory effect on microorganisms such as *Vibrio vulnificus* and *Vibrio parahaemolyticus.* At a final concentration of 0.77 µM, it significantly inhibited the growth of Gram-negative bacteria, including *E. coli* Top10, *V. vulnificus, V. parahaemolyticus*, and *Vibrio alginolyticus* [12]. In the present study, the protein extracts containing ALFPm3 extracted from the culture supernatant showed high bacterial inhibitory activity for more than 24 h. The 24 h inhibition rates of ALFPm3 secreted by T-JiA I3 reached 113.24%, 116.18%, 97.64%, and 98.38% for *Vibrio harveyi*, *Vibrio anguillarum*, *V.algolyticus*, and *V. parahaemolyticus*, respectively. Additionally, the inhibition rate of ampicillin (2 mg/mL) showed a decreasing trend after 8 h, and the 24 h inhibition rates to *V. harveyi, V. anguillarum, V. algolyticus*, and *V. parahaemolyticus* have been reduced to −0.69%, −12.77%, −9.90%, and 8.48%, respectively. Therefore, the ALFPm3 secreted by T-JiA I3 has a longer-lasting inhibition effect than ampicillin, which means that the amount of antimicrobial peptide can be reduced to reduce the cost further. Interestingly, our results showed that the extracellular protein extraction of T-JiA I3 possessed the ability to inhibit common aquaculture pathogenic bacteria, even when diluted 8–80 times. According to the references, the lipopolysaccharide (LPS) structural domain of antilipopolysaccharide factor 3 can effectively bind to the LPS of Gram-negative bacteria and the lipteichoic acid (LTA) of Gram-positive bacteria, preventing the bacteria from further growth through the disruption of their membrane structure [10,40,41].

Compared to other studies focused on extracellular expression, there are many advantages of the work carried out in the present paper. *Pichia pastoris* was used to secrete human antimicrobial peptide LL37. The results showed that LL37 was mainly present in the peroxisomes of cell debris, and in about 62%, only about 19.6% of the target protein was found in the supernatant [42]. Based on De-Mei Meng studies, *P. pastoris* successfully expressed and secreted many antimicrobial peptides rPaDef, and the secretion amount was only 0.087 μg/μL [43]. In the present work, the concentration of the extracellular protein extract of T-JiA I3 is up to 0.91 μg/μL, which is higher than that of similar studies. Additionally, most of the ALFPm3 were kept in the cells, larger than that outside of the cells. It is necessary to improve the secretion efficiency of ALFPm3 in *C. reinhardtii*. Even so, the choice of the extracellular expression of antimicrobial peptides will greatly simplify the protein purification process and reduce the production cost. ALFPm3 with significant antibacterial activity can be obtained through simple centrifugation collection and freeze-drying. This simple process and its easy operation will help promote the application of microalgae-expressed antimicrobial peptides in the aquaculture industry.

In this work, according to protein immunoblot analysis, ALFPm3 was found both intracellularly and extracellularly. It means that not all ALFPm3 is secreted extracellularly, which is similar to the results of previous studies. Hence, the secretory expression of *C. reinhardtii* still needs to be improved by finding a more efficient signal peptide to secrete ALFPm3 efficiently. 

## 5. Conclusions

In this study, we obtained transgenic *C. reinhardtii* T-JiA that could produce and secrete ALFPm3 extracellularly. Its culture supernatant showed an inhibition rate of 97% against four common aquaculture pathogenic bacteria, including *Vibrio harveyi*, *Vibrio anguillarum*, *Vibrio alginolyticus*, and *Vibrio parahaemolyticus*. The highest inhibition rate of 116.18% was observed on the test against *V. anguillarum*. More excitingly, the MIC of the extracts from T-JiA3 to *V. harveyi, V. anguillarum, V. alginolyticus*, and *V. parahaemolyticus* were 0.11 μg/μL, 0.088 μg/μL, 0.11 μg/μL, and 0.011 μg/μL, respectively. This study will support the foundation of the expression of high-activity anti-lipopolysaccharide factors using the secretory expression system in *C. reinhardtii*, providing new ideas for the expression of high-activity AMPs. This strategy simplifies the protein purification process and reduces the production cost, and the bacterial inhibitory activity of ALFPm3 was encouraging. Due to the fact that some ALFPm3 was still stored in the cells of *C. reinhardtii*, signal peptides that can efficiently secrete ALFPm3 is looking promising in terms of promoting the microalgal expression of antimicrobial peptides for application in the aquaculture industry.

## Figures and Tables

**Table 1:** Primer sequences used in this study.

Name	Sequence (5′-3′)	Target Gene	Expected Product (bp)
psaD-P	GGGAATTGGAGGTACGACCGAGAT	IBP1-ALFPm3	684
psaD-T	AGCTCCGATCCCGTATCAATCAGC		
Fi	ATGCCGTCGAGCAGCATGAAGCT	IBP1-ALFPm3	216
ALF3R2	ACATGCGGCCCTTGTAGTACACCT		
actin-F	ACCCCGTGCTGCTGACTG	β-actin	351
actin-R	ACGTTGAAGGTCTCGAACA		

## Data Availability

No new data were created or analyzed in this study. Data sharing is not applicable to this article.
